# Ether‐Linked Glycerophospholipids Are Potential Chemo‐Desensitisers and Are Associated With Overall Survival in Carcinoma Patients

**DOI:** 10.1111/jcmm.70277

**Published:** 2024-12-19

**Authors:** Yu‐Ting Su, Wei‐Chun Chang, Lumin Chen, Ying‐Chun Yu, Wen‐Jen Lin, Jheng‐You Lin, Wei‐Chung Cheng, Juan‐Cheng Yang, Yao‐Ching Hung, Wen‐Lung Ma

**Affiliations:** ^1^ Graduate Institute of Biomedical Sciences, Program for MD/PhD, Research Center for Cancer Biology, School of Medicine China Medical University Taichung Taiwan; ^2^ Department of Obstetrics and Gynecology, Department of Medical Research China Medical University Hospital Taichung Taiwan; ^3^ Department of Obstetrics and Gynecology China Medical University Hospital Hsinchu Branch Hsinchu County Taiwan; ^4^ Department of Obstetrics and Gynecology Asia University Hospital Taichung Taiwan

**Keywords:** alkyl glycerone phosphate synthase, chemosensitivity, ether‐linked phosphatidylethanolamine

## Abstract

Lipid reprogramming in carcinoma is reported to have a role in carcinogenesis, prognosis and therapy response. The lipid reprogramming could be contributed by either autonomous or nonautonomous resources. Since the nonautonomous lipid resources contributed by lipoproteins and their receptors have been reported in epithelial ovarian cancer (EOC), the impact of autonomous lipid metabolites was unknown. This report revealed a unique lipid class, ether‐linked phosphatidyl‐ethanolamine (PE O–), which enhances chemo‐insensitivity and progression in EOC and potentially cross carcinomas. Analysis of CCLEC/GDSCC database and in‐house cell line lipidomes identified PE O– as the major lipid associated with cisplatin/paclitaxel sensitivity. In the testing of PE O– effect on cancer phenotypes, it enhanced cell growth, migratory activities and promoted cisplatin/paclitaxel insensitivity. In addition, treating AGPS inhibitor‐sensitised chemo‐cytotoxic upon cisplatin/paclitaxel treatments. Treating PE O– could reverse AGPS inhibitor chemosensitisation effect on EOC cells. At last, using TCGA‐EOC transcriptome database, the PE O– related gene expressions were positive correlated with patient prognosis in general, or in whom were treated with platin‐ or taxel‐based chemotherapies. The expressions of genes for the synthesis of PE O– aggravates therapy response in EOC patients. PE O– facilitates human carcinoma cell line growth, mobility and chemo‐insensitivity.

## 1 Introduction

Lipids constitute about 15% of cellular dry weight and are involved in modulating fundamental cellular processes. Natural mammalian lipids consist of at least 27 classes, for example, triglycerides, glycerophospholipids, glycolipids, sphingolipids and cholesterol [1]. These biomolecules are not only stored as energy reservoirs but also play important roles in signal transduction [2], immune reaction [3], apoptosis [4] and cell proliferation [5]. Solid tumours share the common feature of uncontrollable proliferation, which creates an anabolic need for lipid metabolism to generate energy, cellular constituents, protein modification or signalling [6]. Alterations in the lipid metabolic pathways are directly driven by successive oncogenic events and ultimately cause cancer cell survival and growth [7, 8].

There are several functional classes of lipids involved in energy metabolism [9, 10] and membrane assembly [9, 11]. Arachidonic acid serves as a substrate for the synthesis of lipids involved in cellular signalling, such as eicosanoids (prostaglandins, leucotrienes, hydroxyeicosatetraenoic acids, epoxide eicosatrienoic acid, hydroperoxyeicosatetraenoic acids), through the cyclooxygenase (COX), lipoxygenase (LOX) and P450 epoxygenase pathways. Dysregulated arachidonic acid metabolism may promote inflammation and tumour‐supporting microenvironments [12]. Another example is the alcohol–lipid metabolites correlated with intracellular reactive oxygen species [13]. Ferroptosis (programmed cell death, regulated by iron‐dependent lipid peroxidation) is the cancer phenotype related to alcohol–lipid metabolism [14].

Carcinomas are cancer cells of epithelial origin and are the most common and lethal human malignancies. Although carcinomas have a mesenchymal origin, they are phenotypically and genotypically distinct [15]. The omics, for example, transcriptome, proteome and metabolome, of human carcinoma cells have been studied and datasets are available in the public domain (CCLEC: Cancer Cell Line Encyclopedia Consortium) [16, 17]. In addition, the chemosensitivity of human carcinoma cell lines has been documented (GDSCC: Genomics of Drug Sensitivity in Cancer Consortium), providing an excellent tool for study [18, 19]. Although lipid metabolites have not yet been explicitly addressed in the databank, the CCLEC and GDSCC databases provide excellent tools for studying lipid metabolic gene expression in association with the chemosensitivity of the ground state.

Epithelial ovarian cancer (EOC) has the highest mortality rate among gynaecological cancers, and its chemoinsensitivity contributes to therapy relapse and high mortality [20, 21]. The varied origins of EOC lead to diverse carcinoma subtypes, including surface epithelial, mucinous, endometrioid and clear cell carcinomas [22]. These EOC subtypes show diverse innate responses (i.e., chemosensitivities) against chemoagent treatments [23]. Whether this diversity is due to genome activity or environmental cues is still under debate. In this study, we used a cell line transcriptome database to examine genome activity aligning with in‐house lipidome data established in our laboratory to detect significant innate chemosensitive lipid biosignatures.

## 2 Materials and Methods

### 2.1 Cell Culture

Sixty‐one cell lines were purchased from the American Type Culture Collection (ATCC) and the Bioresources Collection and Research Center (BCRC), Taiwan Institute of Food Industry Research and Development. Subculture procedures were performed according to the protocols of the ATCC and BCRC. Cells were maintained in various culture media (Table S1) with 10% foetal bovine serum (FBS; Invitrogen), 1% L‐glutamine and 1% penicillin–streptomycin.

### 2.2 IC_50_ Cell Viability

Cell viability under different chemotherapeutic agents was assessed using the WST‐1 assay. Sixty‐one cell lines were seeded into 96‐well plates (5 × 10^3^ or 1 × 10^4^ cells/well) and incubated overnight for attachment. The following day, cells were treated with cisplatin (P4394, Sigma‐Aldrich; ALX‐400‐040, Enzo Life Sciences), paclitaxel (BML‐T104, Enzo Life Sciences), 5‐fluorouracil (F6627, Sigma‐Aldrich), doxorubicin (D1515, Sigma‐Aldrich) or gemcitabine (G6423, Sigma‐Aldrich) for 48 or 72 h. After treatment, WST‐1 reagent (Roche Diagnostics, Laval, Quebec, Canada) was added to each well and incubated for 60 min at 37°C. Absorbance was measured at 450 nm using a microplate reader. IC50 values for each drug were calculated using Prism version 8.0 and Quest Graph IC50 Calculator (AAT Bioquest; https://www.aatbio.com/tools/ic50‐calculator).

### 2.3 Cell Doubling Time

Sixty‐one cell lines (2 × 10^4^ cells each) were seeded onto 6‐well plates (Corning, Midland, NC, USA) and cultured in a 5% CO_2_ humidified incubator at 37°C for 48 h. Cells were detached and harvested using 0.05% trypsin, followed by treatment with 0.4% trypan blue viability dye to exclude dead cells. The viable cells were counted using an automated cell counter (Countess 3 FL, Invitrogen). To calculate the doubling time of each cell line, a growth curve was drawn using the online doubling time calculation free software (http://www.doubling‐time.com/compute.php). The population doubling time formula used was Doubling time = duration * (log (2)/log (final cell concentration) – log (initial cell concentration)), where duration was the cell culture time.

### 2.4 Lipidomics Analysis

Lipid was extracted for mass spectrometry lipidomics with Lipotype GmbH (Dresden, Germany) using a two‐step chloroform/methanol procedure [24, 25]. The extracted lipids were spiked with an internal standard mixture that included cardiolipin 14:0/14:0/14:0/14:0 (CL), ceramide 18:1;2/17:0 (Cer), diacylglycerol 17:0/17:0 (DAG), hexosylceramide 18:1;2/12:0 (HexCer), lyso‐phosphatidate 17:0 (LPA), lyso‐phosphatidylcholine 12:0 (LPC), lyso‐phosphatidylethanolamine 17:1 (LPE), lyso‐phosphatidylglycerol 17:1 (LPG), lyso‐phosphatidylinositol 17:1 (LPI), lyso‐phosphatidylserine 17:1 (LPS), phosphatidate 17:0/17:0 (PA), phosphatidylcholine 17:0/17:0 (PC), phosphatidylethanolamine 17:0/17:0 (PE), phosphatidylglycerol 17:0/17:0 (PG), phosphatidylinositol 16:0/16:0 (PI), phosphatidylserine 17:0/17:0 (PS), cholesterol ester 20:0 (CE), sphingomyelin 18:1;2/12:0;0 (SM) and triacylglycerol 17:0/17:0/17:0 (TAG). After extraction, the organic phase was transferred to an infusion plate and dried using a speed vacuum concentrator. The first‐step dry extract was resuspended in a solution of 7.5 mM ammonium acetate in chloroform/methanol/propanol (1:2:4, V:V:V), while the second‐step dry extract was resuspended in a 33% ethanolic solution of methylamine in chloroform/methanol (0.003:5:1; V:V:V). Liquid handling procedures were executed with the Hamilton Robotics STARlet robotic platform, incorporating the Anti Droplet Control feature for organic solvent pipetting. For acquisition of mass spectrometry (MS) data, samples were directly infused into a QExactive mass spectrometer (Thermo Scientific) equipped with a TriVersa NanoMate ion source (Advion Biosciences). Analysis was performed with both positive and negative ion modes with a resolution of Rm/z = 200 = 280,000 for MS and Rm/z = 200 = 17,500 for tandem mass spectrometry (MS/MS) experiments within a single acquisition. MS/MS was triggered by an inclusion list covering the corresponding MS mass ranges scanned in 1 Da increments. Both MS and MS/MS data were combined to monitor various lipid ions in different adduct forms. The data were analysed using proprietary lipid identification software developed in‐house, which capitalised on the LipidXplorer framework. Subsequent postprocessing and normalisation were performed using an internally developed data management system. For inclusion in further data analysis, only lipid identifications with a signal‐to‐noise ratio > 5 and a signal intensity fivefold higher than that of corresponding blank samples were considered.

### 2.5 Lipid Enrichment and Characteristics Analysis

Using the Lipidsig web tool (http://Chenglab.cmu.edu.tw/lipidsig/) for lipid identification [26], we enriched various lipid features by classifying them as upregulated or downregulated based on log2‐fold change analysis. We then performed an overrepresentation analysis using Fisher's exact test to determine whether a specific class of lipid signature exhibited a significant imbalance among up or downregulated lipid species compared to what would be expected by chance. The cutoff criterion for identifying significantly enriched or missing classes of lipid features was set at a *p* value < 0.05. The MS‐based lipidomic analysis yielded a comprehensive dataset comprising over 2000 individual lipid species and their picomolar abundances. To enhance our understanding of the diversity of lipid functions and characteristics, we analysed two data pipelines based on either lipid species or lipid characteristics. In our analysis of lipid characteristics, we transformed the expression of lipid species into a new table of expression of various characteristics. This process involved categorising and summarising all lipid species into specific variables, such as lipid class, the number of double bonds and carbon chain length. The newly generated table of characteristics served as the basis for subsequent analyses. Furthermore, we explored interactions between various characteristics by combining multiple features, such as chain length and double bond number.

### 2.6 Wound Healing Assay (Migration Assay)

EOC cell lines SKOV3 and OVCAR3 were pretreated with 10 nM ether‐linked phosphatidylethanolamine (PE O–; #878130, Avanti) for 7 days. Subsequently, the cells pretreated with PE O– were seeded into culture inserts (Cat No: 81176, ibidi GmbH, Martinsried, Germany) at a density of 1 × 10^4^ cells per well. After overnight attachment (reaching 90% confluence per well), the culture inserts were removed and conditional media were provided for each experimental group. For instance, the control group received fresh growth medium only, while the PE O– group was continuously supplied with 10 nM PE O– in the growth medium. In the experiment, the chemotherapeutic drug groups, cisplatin (5 μM) and paclitaxel (5 nM), were treated together with PE O–. The initial observation (0 h) was made upon chemical addition. Images were captured at 0 and 24 h under a 100× magnification optical microscope (Nikon Eclipse TS100). Subsequently, a quantitative analysis of the areas was performed using ImageJ software (https://imagej.nih.gov/ij/).

### 2.7 Colony Formation

The EOC cell lines (SKOV3 and OVCAR3) were seeded onto 6‐cm plates (1 × 10^5^ cells/dish) and divided into two groups: one treated with growth medium (DMEM + 5% FBS) as control and the other with 10 nM PE O– (16:0–18:1 PE O–; #878130, Avanti) and 500 μM AGPS inhibitor (Cat#: APL‐AGPS‐IN‐2i, Apolo Biochemical Inc.) in growth medium (DMEM + 5% FBS) for 7 days. Both groups were subcultured into 6‐well plates (500 cells/well) with a culture medium containing 5% FBS. After 2 days of incubation for cell attachment, they were treated with the indicated drugs and incubated for 10 days. The cells were then fixed with 4% formaldehyde solution for 30 min at room temperature, stained with 1% crystal violet overnight, washed with PBS and photographed using an imager (ChemiDoc XRS+ System with Image Lab Software #1708265). Colonies were counted using ImageJ software.

### 2.8 Kaplan–Meier Analysis of Overall Survival of Patients With Ovarian Cancer

The 5‐year overall survival (OS) of patients with EOC was analysed using the Kaplan–Meier Plotter, a web‐based gene survival analyser (http://kmplot.com/analysis/index.php?p=service&cancer=ovar). The 5‐year OS of various EOC cohorts was determined after stratification by median classifier expression levels. The ovarian cancer subtypes included in the analysis were all patients (unclassified; *n* = 1656), patients who received platinum therapy (*n* = 1409) and patients who received paclitaxel therapy (*n* = 793). The input genes for this analysis were ETNK1 (224453_s_at), ETNK2 (219268_at), PCYT1A (CTPCT) (204210_s_at), PCYT2 (230044_at), LGI1 (EPT) (206349_at), GNPAT (201956_s_at) and AGPS (225108_at).

### 2.9 Hazard Ratio Summation to Assess the Target Score of Patients With Ovarian Cancer

In our previous study, we used the Kaplan–Meier Plotter survival analyser to calculate the impact of gene clusters on different GCa conditions [27].

The hazard ratio (HR) scoring formula was as follows:

$$\begin{array}{c} \mathrm{HR}\;\text{score}=\left(\mathrm{Avg}.\;\text{of}\;\mathrm{HR}\;\text{of gene sets}\right)\\ =\frac{\sum \left(\mathrm{HR}n-1\right)\times \left(\log_{10}\left(p-\text{value}\right)\right)}{n}\times 100 \end{array}$$

The HR of each gene was minus one, to adjust the effect of genes, multiplied with the negative log10 (*p*‐value) to balance the importance of genes. The resulting sum was divided by the number of genes and then multiplied by 100 to obtain the HR score, which represented the average HR for each gene. A threshold of 100 was used to indicate the significance of gene clusters. To validate the algorithm's effectiveness, we applied it to the enzymes involved in the CTP‐PE pathway (Kennedy pathway) [28] and ether phospholipid biosynthesis [29]. The gene identifiers used were ETNK1 (224453_s_at), ETNK2 (219268_at), PCYT1A (CTPCT) (204210_s_at), PCYT2 (230044_at), LGI1 (EPT) (206349_at), GNPAT (201956_s_at) and AGPS (225108_at).

### 2.10 Statistical Analysis

Quantitative data were analysed using unpaired Student's *t*‐tests in Prism version 8.0 (GraphPad software) to identify the significant differences between groups and categorical variables. A *p*‐value < 0.05 was considered statistically significant. All values are presented as mean ± SEM. All comparisons were made relative to the controls and the significance of the difference was indicated as ns, not significant, *p < 0.05, **p < 0.01, ***p < 0.001 and ****p < 0.0001.

## 3 Results

### 3.1 Glycerophospholipids Are the Consensus Lipid Metabolites in the Transcriptome and Lipidome of the Human Carcinoma Chemosensitivity Biosignature

**FIGURE 1:**
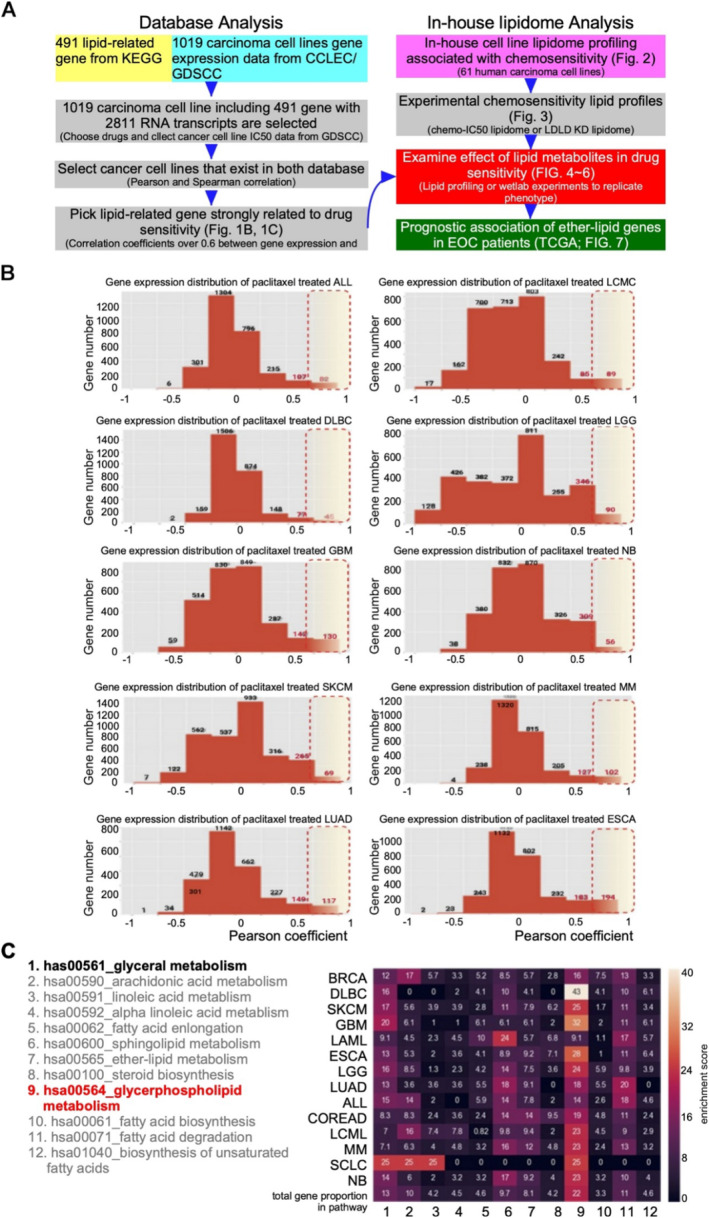
Strong lipid‐related pathway correlations identified in CCLEC and GSDCC databases. (A) The study scheme for combining database analyses (left‐hand side), in‐house lipidome analysis (upper right) and wet‐laboratory verification (red‐coloured background) of the effects of lipid metabolites on chemosensitivity, and then eventual evaluation of data from the TCGA EOC database. (B) Pearson correlation coefficients (Y‐axis) indicated a significant correlation between lipid‐related gene expression (x‐axis) and IC_50_ in paclitaxel‐treated cell lines. The different TCGA cancer‐type classifications and chosen cancer types with more than five samples for plotting bar graph. We calculated the Pearson correlation coefficient for the relationship between each transcript and IC_50_ for every cancer type. The red‐dashed circle denotes a correlation coefficient > 0.7. When the Pearson correlation coefficient is > 0.7, the number of transcripts is labelled in red. These transcripts are lipid‐related genes. The horizontal axis represents the Pearson correlation coefficient, while the vertical axis represents transcript count. (C) We selected the transcripts with a Pearson correlation coefficient of > 0.7 for each cancer type and found the lipid‐related pathways for KEGG. Then, we counted the number of transcripts for each pathway for all cancer types. All experiments are from at least three reproducible experiments. ALL, acute lymphocytic leukaemia; BRCA, breast invasive carcinoma; COREAD, colorectal adenocarcinoma; DLBC, diffuse large B‐cell lymphoma; ESCA, oesophageal carcinoma; GBM, glioblastoma; LAML, acute myeloid leukaemia; LCMC, lung cancer mutation consortium; LGG, low grade glioma; LUAD, lung adenocarcinoma; MM, multiple myeloma; NB, neuroblastoma; SCLC, small cell lung cancer; SKCM, skin cutaneous melanoma.

Our aim was to resolve chemosensitivity‐related lipid metabolites via alignment between the public carcinoma transcriptome and in‐house lipidome databases and then validate the biological phenotype in EOC cells. The study scheme is illustrated in Figure 1A.

In the chemosensitivity‐related lipid gene analysis, genome activity related to lipid metabolism was investigated using the CLECC and GDSCC databases. First, we selected lipid metabolism gene IDs from KEGG (491 gene) and downloaded transcriptome data from the CLECC and GDSCC databases (1091 cell lines). We identified 2611 mRNA transcripts. Then, gene expression levels were associated with IC_50_ of the five chemoagents, that is, 5‐fluouracil (5‐FU), cisplatin, paclitaxel, doxorubicin and gemcitabine. Because paclitaxel is the commonly used first‐line chemoagent for EOC, its chemosensitivity was selected to demonstrate the association with lipid‐related genome activity (Pearson correlation coefficient > 0.7). The association score of gene numbers of various human carcinoma types is presented in Figure 1B. We chose several cancer types, for example, ALL, DLBC, GBM, SKCM, LUAD, LCMC, LGG, NB, MM and ESCA, as examples to show the high positive correlation gene numbers. The high positive correlation genes were selected and subjected to further lipid pathway analysis (Table S2). We calculated the proportion of gene numbers in each KEGG lipid metabolic pathway (Figure 1C). For each cancer type (each lane), the number in the box indicated the proportion of genes in the total high correlation score genes. The colour of each square, from dark to light, indicated increasing enrichment scores corresponding to each lipid pathway (among rows 1–12). From the two dimensions (proportion in carcinoma type and correlation score in pathways), glycerol‐backboned phospholipid metabolism was found to be the consensus pathway in various carcinomas (Figure 1C).

**FIGURE 2:**
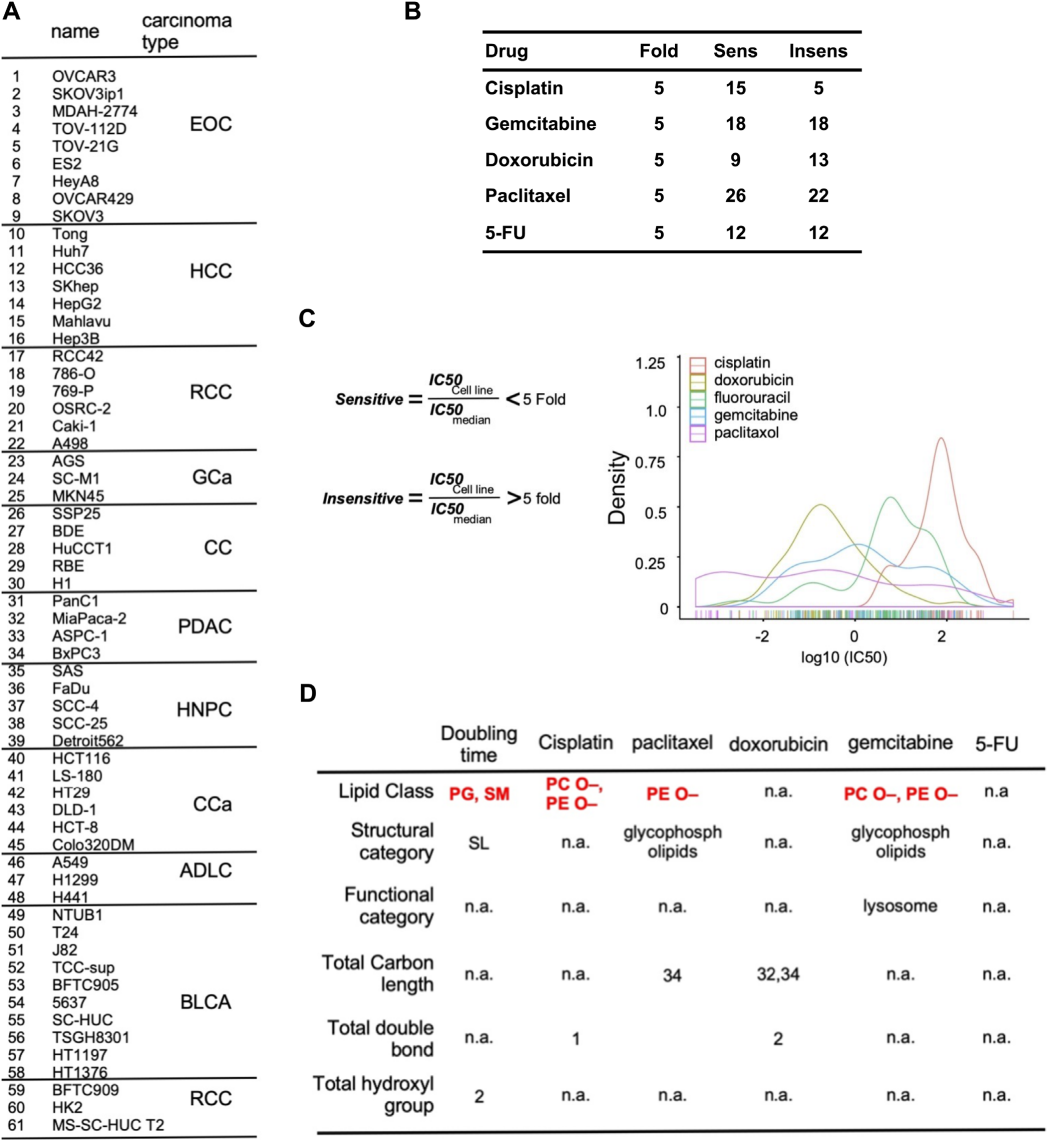
Database analysis in conjunction with an in‐house lipidome analysis for exploring candidate lipids. (A) In‐house cell line databank established in the principal investigator's (PI's) and co‐PI's laboratory for proposed studies. (B) Definition of sensitivity or resistance threshold based on fivefold changes in IC_50_ of the five drugs. (C) Documentation of the IC50 values of five chemotherapeutic drugs for the in‐house cell lines was conducted to distinguish between chemosensitive and chemoinsensitive lines, along with a distribution analysis of the IC50 values for each drug across the cell lines. (D) Integration table of lipidomic analyses in various categories, for example, lipid class, structure category, functional category, total carbon‐chain length, total double‐bond and total hydroxyl group. The analytic categories are associated with various variables, for example, doubling time of cell growth and five chemoagents IC_50_. All experiments are from at least three reproducible experiments.

The cell line list in the in‐house cell line lipidomic analysis is shown in Figure 2A. The cell line identifiers for chemosensitive and chemoinsensitive responses to the five chemoagents are shown in Figure 2B. The five chemoagents, cisplatin, doxorubicin, fluorouracil (5‐FU), gemcitabine and paclitaxel, are commonly used in cancer therapy. The doubling time of each cell line was also measured for the consensus mechanism of action of the five chemoagents' blockade of the cell cycle. We performed baseline lipidome profiling of 61 cell lines. The definitions of chemosensitive and chemoinsensitive cell lines were based on the IC_50_ of individual lines divided by the median IC_50_ of all 61 cell lines. Cells with a fivefold lower value were defined as sensitive, whereas those with a fivefold higher value were defined as insensitive. The IC_50_ of the five chemoagents for the 61 cell lines are shown in Figure 2C. The lipid classes or characteristics in the comparison between chemoinsensitive and chemosensitive cells are summarised in Figure 2D. Among lipid profiles associated with chemosensitivity, ether‐linked phospholipids were the consensus for cisplatin, paclitaxel and gemcitabine, but not for doxorubicin and 5‐FU. Interestingly, lipid metabolites of common cell characteristics for the mechanism of action, doubling time of cell growth, were not related to ether lipids, but were related to phosphatidylglycine (PG) and sphingomyline (SM). For other lipid features, for example, structural category, functional category, double‐bond numbers and hydroxyl group numbers, there was no consensus between chemosensitive and chemoinsensitive cell lines.

In brief, the consensus innate chemosensitivity lipid metabolites from the integrated transcriptomic analyses of the CCLEC and GDSCC databases and lipidomic analyses of the 61 in‐house cell lines from the lipidome database were ether‐linked glycerophospholipids.

### 3.2 PE O– Is the Dominant Lipid Positively Correlated With the Chemoinsensitivity of EOC Cells

**FIGURE 3:**
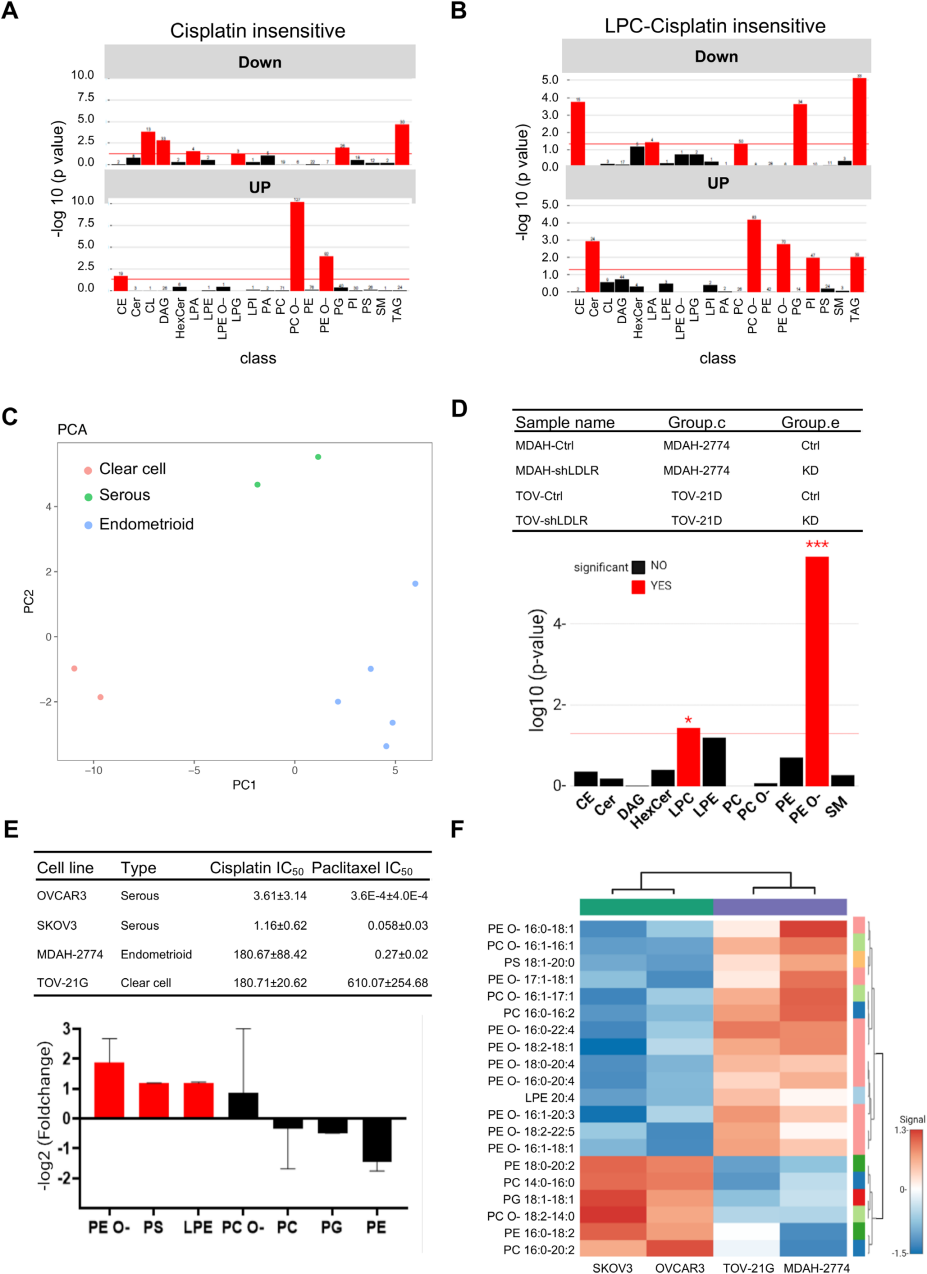
Enriched ether‐linked phosphatidylethanolamine (PE O–) represents the dominant chemosensitivity biosignature in insensitive epithelial ovarian cancer (EOC) cells. (A) Lipidomic enrichment of 5 cisplatin‐insensitive and 15 cisplatin‐sensitive cell lines. PE O–, ether‐linked phosphatidylcholine [PC O–] and cholesterol ester [CE] levels are elevated in the 5 cisplatin‐insensitive cell lines. (B) Lipidomic enrichment of cisplatin‐loaded liposomal nanoparticle drug (LCLND)‐treated cisplatin‐insensitive cells revealed upregulation of ceramide, PC O–, PE O–, phosphatidylinositol [PI] and triacylglycerol [TAG]. (C) Principal component analysis (PCA) analysis was performed for various EOC cell lines and revealed three distinct groups: Serous, clear cell and endometrioid types. Samples are colour‐coded based on their cell type. (D) Lipidomic data for MDAH‐2774 and TOV‐21G parental (par) cells are compared with their low‐density lipoprotein receptor (LDLR) knockdown (KD). Two lipids, lysophosphatidylcholine (LPC) and PE O–, were significantly enriched. LPC was upregulated and PE O– was downregulated in KD groups. (E) PE O– was significantly enriched in insensitive EOC cell lines (lipid expression of insensitive cells [MDAH‐2774, TOV‐112D] divided by that of sensitive cells [SKOV3, OVCAR3]: ~ twofolds). The PE O– level was increased in resistant EOC cell lines. (F) Heat‐map lipid profiling: Sensitive (SKOV3, OVCAR3) versus insensitive (MDAH‐2774, TOV‐112D) EOC cell lines. The spectrum from blue to red indicates the variation in lipid species among cells. All experiments are from at least three reproducible experiments. CE, cholesterol ester; Cer, ceramide; CL, cardiolipin; DAG, diacylglycerol; HexCer, hexosylceramide; LPA, lyso‐phosphatidate; LPC, lysophosphatidylcholine; LPE O–, ether‐linked lysophosphatidylethanolamine; LPE, lyso‐phosphatidylethanolamine; LPG, lyso‐phosphatidylglycerol; LPI, lyso‐phosphatidylinositol; PA, phosphatidate; PC, phosphatidylcholine; PCO–, ether‐linked phosphatidylcholine; PE O–, ether‐linked phosphatidylethanolamine; PE, phosphatidylethanolamine; PG, phosphatidylglycerol; PI, phosphatidylinositol; PS, phosphatidylserine; SM, sphingomyelin; TAG, triacylglycerol.

Our team previously studied the effects of lipids on chemosensitivities from various perspectives [30, 31]. Here, we enlisted four hypothesis‐driven lipidomic analyses of chemosensitivity using the LipidSig lipidomics analysis platform [26] (http://chenglab.cmu.edu.tw/lipidsig/). (1) Naïve cisplatin chemosensitivity lipidome (Figure 3A): 61 cell lines divided by cisplatin IC_50_ into high (chemoinsensitive) versus low (chemosensitive). (2) LPC–cisplatin insensitive lipidome (Figure 3B): lysophosphatidylcholine (LPC) was the cisplatin sensitiser [31], with LPC–cisplatin representing the LPC‐coated liposome nanoparticle encapsulating cisplatin. (3) LDLR‐mediated chemoinsensitivity [30] (Figure 3C): the LDLR knockdown resulted in cisplatin sensitisation. The lipidomes of LDLR knockdown and LDLR parental EOC cells were compared. (4) Native EOC subtype comparison (Figure 3D–F): the difference in chemosensitivity between the serous and the endometrioid subtypes of EOC is well known, with the serous subtype being more sensitive to platin treatments [30]; therefore, the lipidome profiles of two cell lines from each subtype were compared.

In the first comparison (cisplatin chemosensitivity lipidome of 61 cell lines), three lipid classes were upregulated in the comparison of chemoinsensitive and chemosensitive lipidomes, for example, ether‐linked phosphatidylcholine (PC O–), PE O– and cholesterol ester (CE) (Figure 3A), with PC O– and PE O– being more significant. In the second comparison, we compared the lipidome profile between LPC–cisplatin liposome nanoparticle drug (LCLND)‐treated cisplatin‐insensitive cells and resistance cells (Table S3). The results showed that ceramide, PC O–, PE O–, PI (phosphatidyl‐inositol) and TAG (triacylglycerol) were upregulated in the lipidomes of the insensitive group (Figure 3B). Finally, we profiled and analysed the lipidome of the EOC subtypes, for example, clear cell, serous and endometrioid. The PCA analysis found that the three subtypes differed in their lipidome patterns (Figure 3C). In the third comparison, we compared the lipidomes of LDLR knockdown (sensitive) and LDLR parental (Ctrl; insensitive) endometrioid EOC cells, for example, MDAH‐2774 and TOV‐112D cells. The results shown in Figure 3D indicated that LPC was significantly elevated, while PE O– was significantly reduced. Therefore, PE O– was the cisplatin desensitiser in the dataset. While ranking lipid metabolites in EOC cells by comparing paclitaxel‐insensitive to paclitaxel‐sensitive, we found that PE O– was the most upregulated (Figure 3E). In detailed profiling of insensitive and sensitive, the enlisted lipid species were mostly PE O– upregulated (Figure 3F).

In brief, the four hypotheses‐driven lipidomic analyses of the in‐house lipidome database revealed that PE O– was the consensus lipid metabolite corresponding to paclitaxel insensitivity.

### 3.3 PE O– Promotes Malignant Phenotypes and Paclitaxel Insensitivity in EOC Cells

**FIGURE 4:**
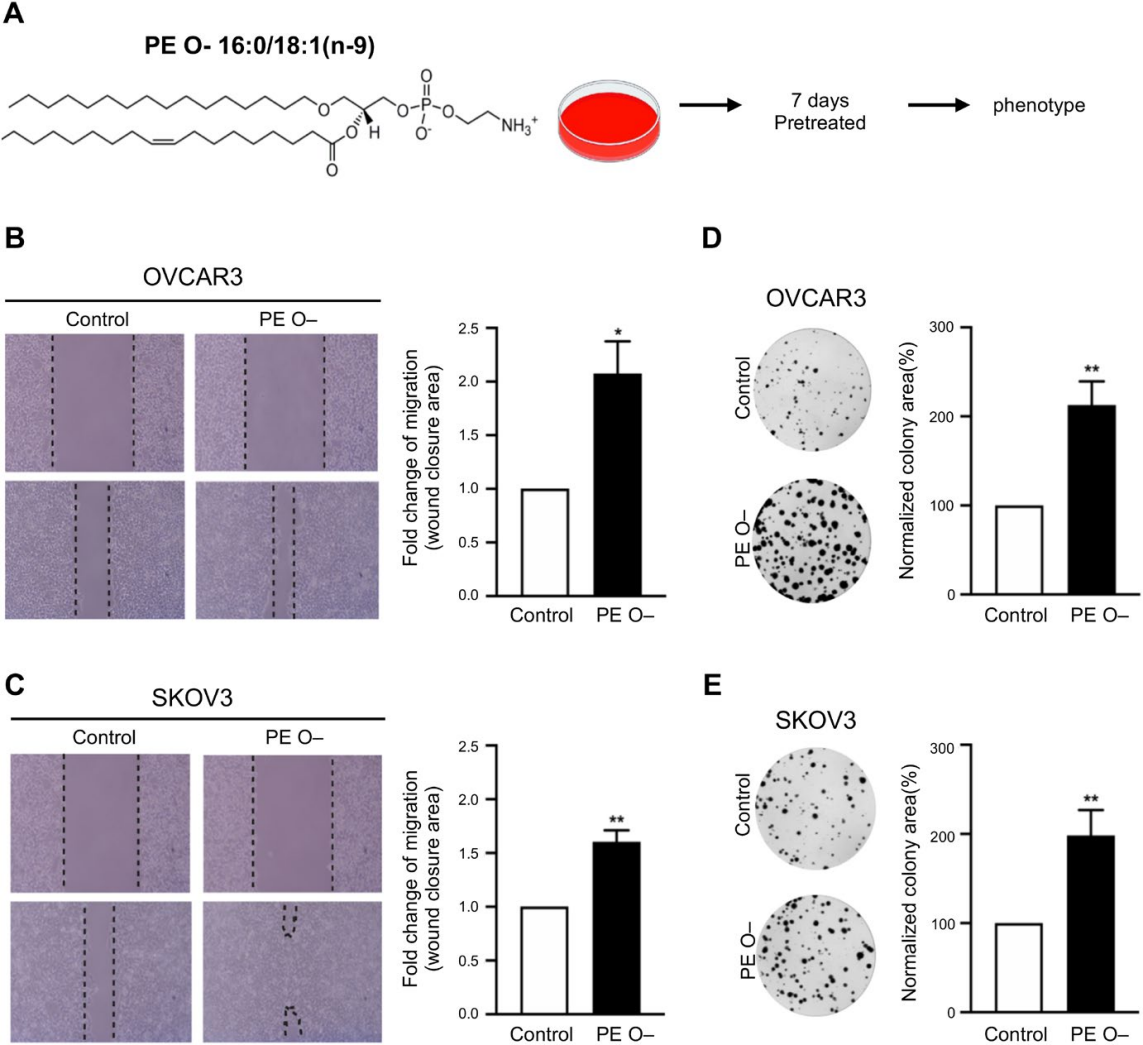
Ether‐linked phosphatidylethanolamine (PE O–) supplementation boosts tumour migration. (A) Structural formula of PE O– (18:1 (n9)‐16:0) and schematic representation of cells pretreated with 10 nM PE O– or untreated before the functional assay. For the pretreatments, cells were seeded onto 6‐cm dishes following the standard subculture procedure. The PE O– medium (including 10 nM PE O– in DMEM with 5% FBS) or the control medium (DMEM with 5% FBS) was applied as indicated for 7 days. (B, C) OVCAR3 and SKOV3 cells pretreated with PE O– showed higher migratory capacity. The left part of (B, C) depicting the line graph shows the cell migration distance at 0 h (upper panel) and 24 h (lower panel). The bar graph on the right shows fold change information for the treatment groups compared to that for the control group. The migrating area (%Area of the view) was determined over two independent fields of view at 100× magnification and averaged for each replicate. (D, E) The results of colony formation showed the growth ability of OVCAR3 and SKOV3 cells treated with PE O– for 10 days. OVCAR3 and SKOV3 cells showed higher colony formation after pretreatment with PE O–. The bar graph on the right represents fold change information for the treatment groups compared to that for the control group. The bar chart presents the mean ± SEM of three experiments. Significant changes are indicated by * (*p* < 0.05) and ** (*p* < 0.01).

Our analysis strongly recommended PE O–as a chemosensitiser (Figures 1, 2, 3). In order to test the causal relationship between PE O– and chemosensitivity in EOC cell lines, two serous‐subtype EOC cell lines, that is, SKOV3 and OVCAR3, were tested in vitro. The most upregulated PE O– (O‐; PE:16:0:0–18:1:0) species was selected to pretreat EOC cells for 7 days (Figure 3F), and then observe cellular phenotypes, for example, growth or mobility (Figure 4A). In the case of cell migration capacity, the PE O– treatment significantly facilitated wound healing activity in both OVCAR3 (Figure 4B) and SKOV3 (Figure 4C) EOC cells. In addition, PE O– treatment also significantly facilitated colony‐forming activity in OVCAR3 (Figure 4D) and SKOV3 (Figure 4E) EOC cells.

**FIGURE 5:**
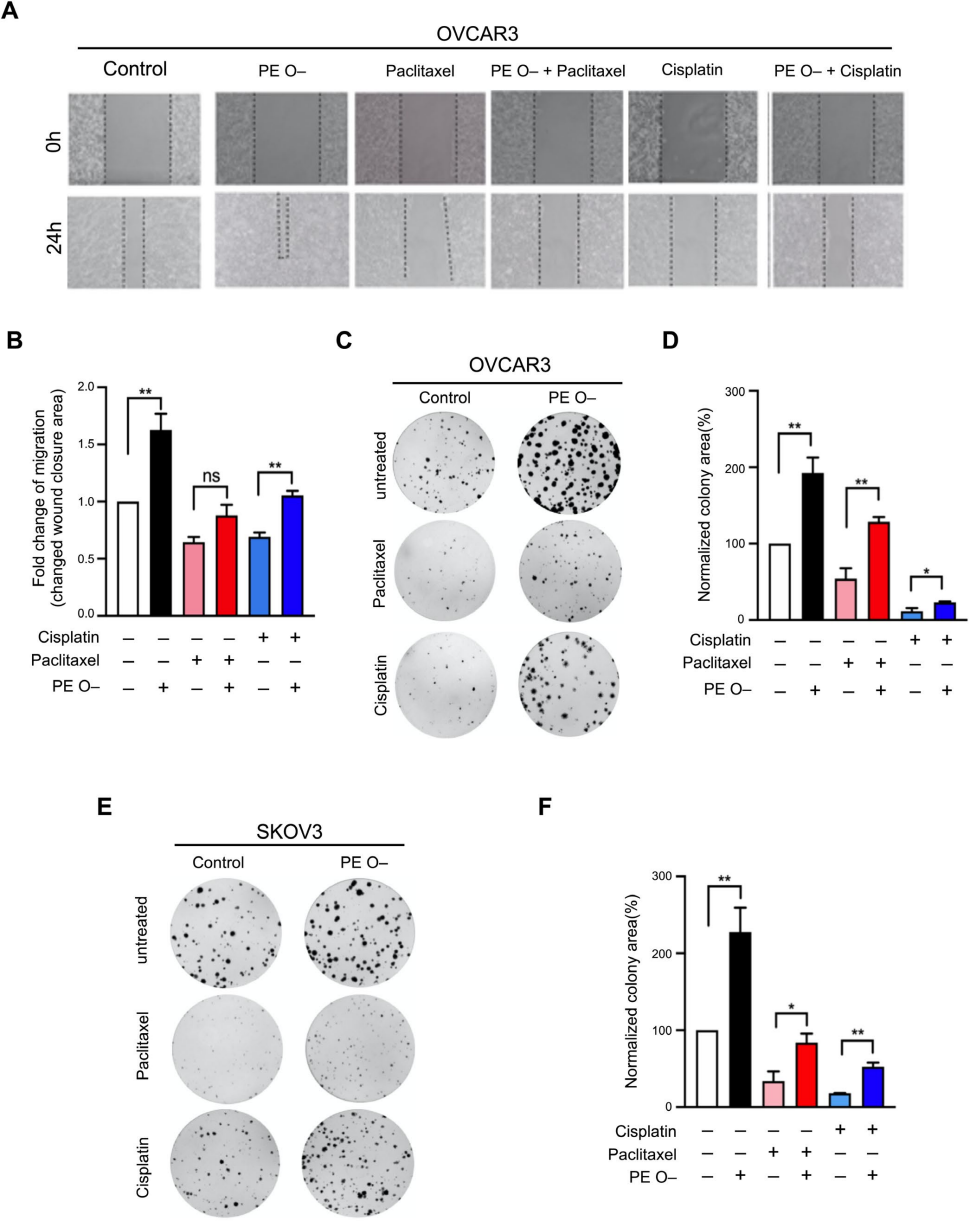
Ether‐linked phosphatidylethanolamine (PE O–) can desensitise cisplatin/paclitaxel cytotoxicity. (A) OVCAR3 cells pretreated with PE O– exhibited enhanced in vitro migration, which conferred resistance to the cytotoxic effects of cisplatin and paclitaxel. Photographic images were captured at 0 and 24 h. (B) Migration capacity over time (24 h) was quantified by measuring the percentage of migrated area. Data are presented as the percentage of migrated area relative to the area at 0 h, normalised to the control (untreated). (C) OVCAR3 cell lines were treated as follows: Control (growth medium), cisplatin (5 μM), paclitaxel (5 nM) and combined treatment (cisplatin plus PE O– or paclitaxel plus PE O–). The results of the colony formation assay indicated that OVCAR3 cells were resistant to drugs under PE O– treatment. (D) Data are presented as % of colony area compared to the control group and normalised with the control group (no treatment). (E) SKOV3 cell lines underwent various treatments, including control (growth medium), cisplatin (5 μM), paclitaxel (5 nM) and combined treatments (cisplatin plus PE O– or paclitaxel plus PE O–). The colony formation assay results revealed the development of drug resistance in SKOV3 cells following PE O– treatment. (F) The data are expressed as the percentage of colony area relative to the area in the control group and normalised to the untreated control group. Data are presented as mean ± SEM of three experiments. Significant changes are denoted as ns, not significant, *(*p* < 0.05) and **(*p* < 0.01).

To investigate the facilitation of chemosensitivity by PE O– in EOC cells, we tested cisplatin or paclitaxel, co‐treated with PE O–, to observe cellular phenotypes, for example, migration and growth. First, we examined the sensitivity of two EOC cell lines (OVCAR3 and SKOV3; high‐grade and low‐grade serous EOC subtypes respectively) against cisplatin and paclitaxel. In the case of cell mobility of chemoinsensitive phenotypes, PE O– facilitated cell migration, even after paclitaxel or cisplatin treatment of OVCAR3 cells (Figure 5A,B). In the case of the colony‐forming capacity of chemoinsensitive phenotypes, PE O– promoted cell growth even after paclitaxel or cisplatin treatment of OVCAR3 (Figure 5C,D) or SKOV3 cells (Figure 5E,F).

**FIGURE 6:**
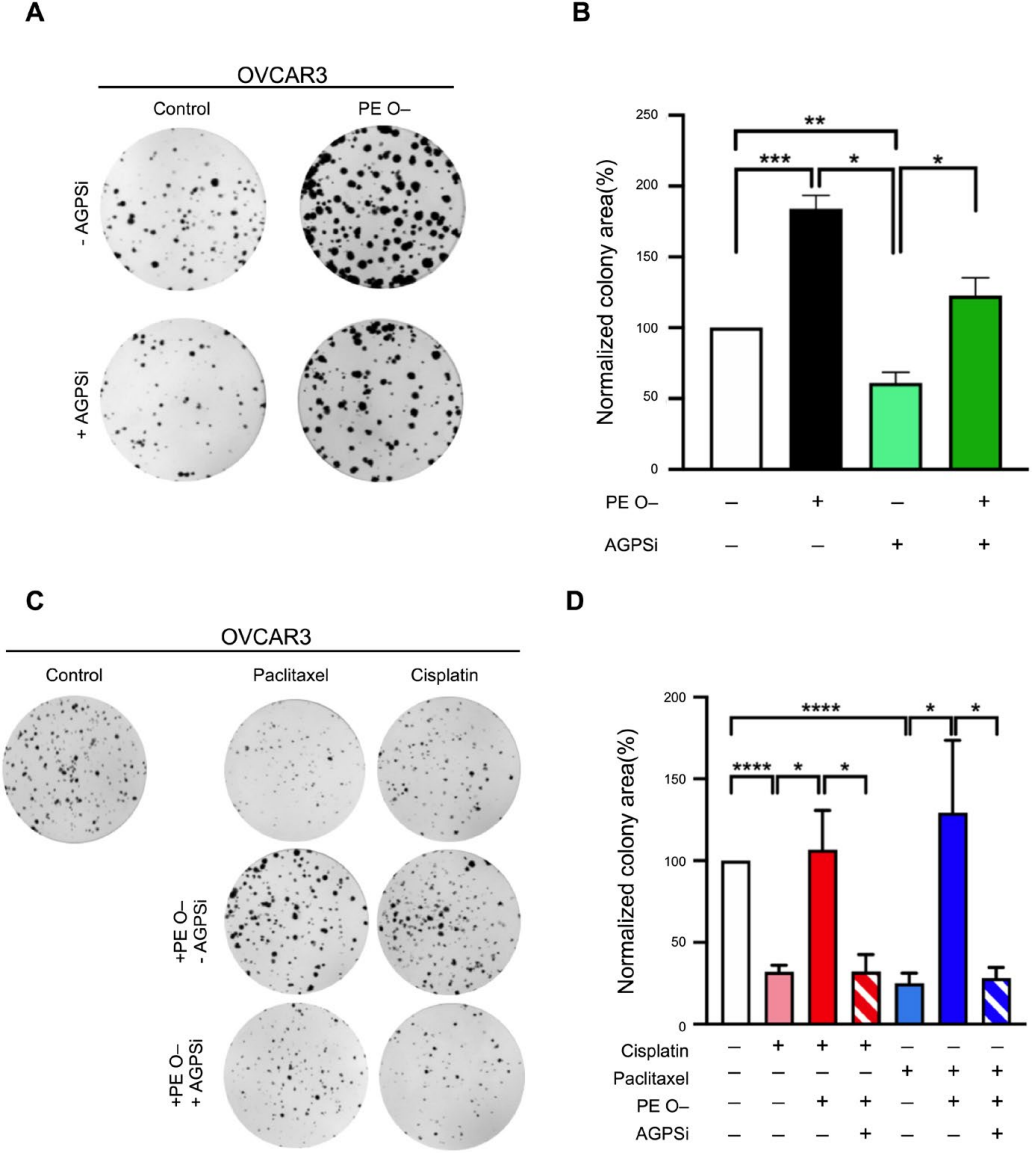
Ablation of ether‐linked phosphatidylethanolamine (PE O–) with AGPS inhibitor could sensitise cisplatin/paclitaxel cytotoxicity of chemosensitive epithelial ovarian cancer (EOC) cells. (A) Representative images of colony formation assay following treatment with AGPSi (500 μM), PE O– (10 nM) or a combination of the two. OVCAR3 cells were seeded into 6‐well plates with a density of 500 cells per well. Chemicals as indicated were applied consecutively for 10 days. (B) Quantification of colony formation area (%Area). (C) Representative results for colony formation, showing the growth ability of OVCAR3 cells with different treatments for 10 days. Cells were treated with chemo‐reagents alone (5 μM cisplatin or 5 nM paclitaxel individually), chemo‐reagent combined with PE O– and triple‐combination (including PE O– and AGPSi). (D) Quantification of colony formation. Data are presented as mean ± SEM of three independent experiments. Statistical significance is shown as ns, not significant, *p < 0.05, **p < 0.01, ***p < 0.001 or ****p < 0.0001 versus the vehicle control group. As indicated by the relative level of colony formation, cisplatin and paclitaxel had a cytotoxic effect that could be reversed by co‐treatment with PE O–, which meant that PE O–significantly increased the proliferation ability of chemosensitive cells. Until application of the triple reagents, AGPS‐IN‐2i could restore chemosensitivity by ablating PE O–.

The purpose of this study was to discover lipid metabolites responsible for innate chemosensitivity. After we found that PE O– was a malignant cellular phenotype and chemoinsensitivity facilitator, the next step was to define whether endogenous production of PE O– was critical for cellular phenotypes. In order to achieve this goal, an AGPS inhibitor (AGPSi) [32] was added to the treatment procedure to observe colony‐forming capacity. Treatment with AGPSi alone reduced the colony area on the culture plates by approximately 40% compared to the control. However, when combined with PE O–, the effect of AGPSi on OVCAR3 cells was reversed, increasing the colony area by about 60% (Figure 6A,B). Treatment with paclitaxel and cisplatin alone reduced the colony area of OVCAR3 cells by approximately 70% and 75%, respectively, compared to the control. When PE O– was combined with paclitaxel or cisplatin, the colony area increased by about 70% and 100%, respectively, compared to the chemotherapy‐only groups. Additionally, combined treatment with AGPSi and chemotherapy + PE O– resulted in a further reduction in colony area by approximately 40% and 70%, respectively (Figure 6C,D), confirming that AGPS inhibitor treatment sensitises cancer cells to the cytotoxic effects of cisplatin and paclitaxel.

To summarise the conclusions in Figures 4, 5, 6, endogenous PE O– production by AGPS chemodesensitised the serous subtype of EOC cell lines against cisplatin and paclitaxel challenge.

### 3.4 Ether Lipid‐Synthesising Enzymes Are Prognostic Markers of EOC and Potential Targets for EOC Therapeutics

**FIGURE 7:**
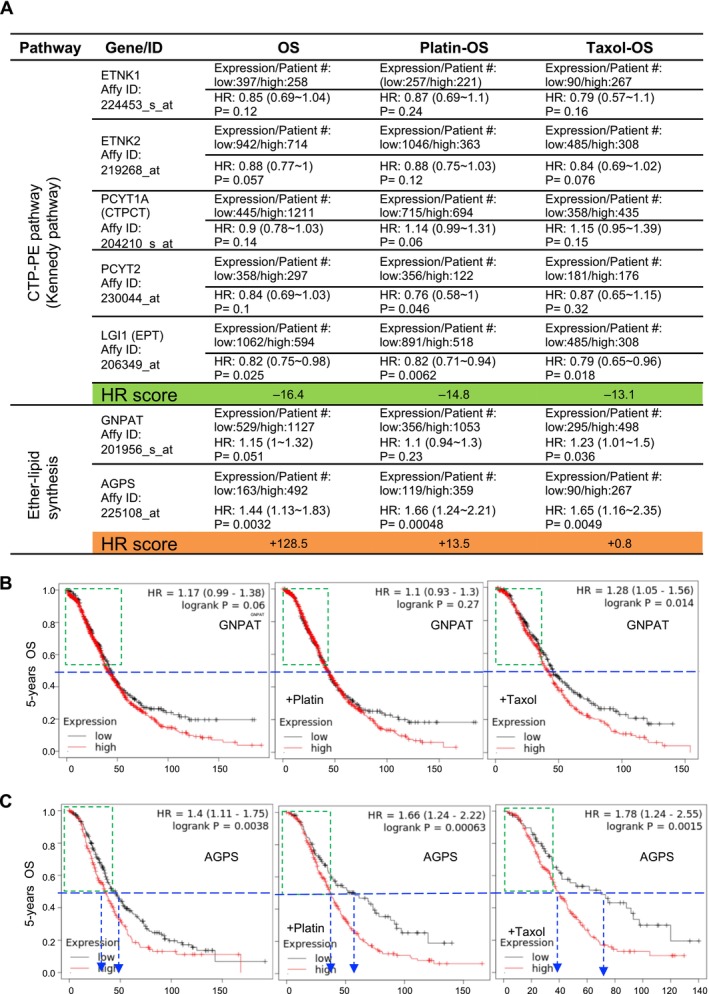
Five‐year overall survival (OS) of patients with epithelial ovarian cancer (EOC) assessed using the Kaplan–Meier plotter for ether phospholipid enzymes, including GNPAT and AGPS. (A) The hazard ratio (HR) was calculated for the OS of patients with EOC progression after treatment with ether phospholipid biosynthetic enzymes with different therapies (nonchemotherapy, platinum therapy and paclitaxel therapy). The enzymes related to ether phospholipids in the CTP‐PE pathway (Kennedy pathway) are ETNK1, ETNK2, PCYT1A, PCYT2 and LGI1 (EPT). The enzymes related to ether phospholipids in ether lipid synthesis are GNPAT and AGPS. (B) GNPAT expression status in untreated, platinum‐treated and paclitaxel‐treated patients shows the following results. The HR for GNPAT of untreated patients was 1.15 (range 1–1.32; *p*‐value = 0.051). The HR for platinum‐treated GNPAT was 1.1 (range 0.94–1.3; *p*‐value = 0.23). The HR for paclitaxel‐treated GNPAT was 1.23 (range 1.01–1.5; *p*‐value = 0.036). (C) AGPS expression status in untreated, platinum‐treated and paclitaxel‐treated patients shows the following results. The HR for AGPS in untreated patients was 1.44 (range 1.13–1.83). For platinum treatment, the AGPS HR was 1.66 (range 1.24–2.21; *p*‐value = 0.00048). For paclitaxel treatment, the AGPS HR was 1.65 (range 1.16–2.35; *p*‐value = 0.0049).

There are two pathways for synthesising PE and PE O– in the cells. One is CTP‐PE synthesis pathway (also known as the Kennedy pathway) for producing cellular PE [33], and the other is ether lipid synthesis for producing ether phospholipids. These two pathways are distinct without known direct up‐ or downstream regulatory relationships. The impacts of these two pathways on EOC prognosis have not been investigated previously. In light of the discovery of the chemoinsensitive role of endogenous PE O– synthesis described in previous sections, we further examined the effect of gene expression in association with EOC prognosis of various clinical features, for example, platin or taxol treatment. The enzyme cascade of synthesis of phosphatidylethanolamine from ethanolamine involves several important enzymes, for example, ETNK1/2 (ethanolamine kinase 1/2) [34], PCYT1A/2 (putative choline‐phosphate cytidylyltransferase; choline‐phosphate cytidylyltransferase, also known as CTPCT [CTP: phosphocholine cytidylyltransferase]) [35], and LGI1 (leucine‐rich, glioma‐inactivated 1; also known as ethanolaminephosphotransferase [EPT]) [36]. Two main enzymes participate in the process of synthesis of ether lipids—GNPAT (glyceronephosphate O‐acyltransferase [37]) and alkylglyceronephosphate synthase (AGPS; [38]). The HR and p‐value of the impact of gene expression on OS in ovarian cancer were evaluated using the Kaplan–Meier Plotter [39] web‐tool platform (Figure 7A). We implemented an established method of evaluating genes with pathway impact on cancer prognosis [27] to examine the effects of CTP‐PE synthesis and ether lipid synthesis pathways on the OS of patients with ovarian cancer calculated as HR. The HR of OS in general was −16.4 for CTP‐PE synthesis and +129.5 for ether lipid synthesis. The HR of the OS of platin‐treated patients was −14.8 for CTP‐PE synthesis and +11.5 for ether lipid synthesis. The HR of the OS of taxel‐treated patients was −13.1 for CTP‐PE synthesis and +2.0 for ether lipid synthesis. This result indicated that the synthesis of ether lipid had a more positive impact on ovarian cancer prognosis, whereas PE synthesis was less important. In order to further analyse the importance of the ether lipid synthesis gene, GNPAT and AGPS were compared side by side under different ovarian cancer conditions. There was no significant survival difference between high and low GNPAT expression in patients (Figure 7B). The survival trends were almost identical in the first half of the observation period (~40 months). However, survival was significantly poor under high AGPS expression compared to low expression (Figure 7C). The survival impact of AGPS expression was initiated in the first half of the observation period, particularly for platin (from 40 to 50+ months) and taxol (from 40 to ~70 months) therapies. In summary, the TCGA analysis of endogenous PE O– synthesis revealed that AGPS might be critical for ovarian cancer chemotherapy‐related prognosis.

## 4 Discussion

The results of this study lead to three conclusions. First, we discovered that in unbiased approaches, ether‐linked glycerophospholipids were the dominant lipid class associated with innate chemoinsensitivity. The integration of analyses of publicly available transcriptomic data and in‐house lipidomic data narrowed down PE O– as the consensus lipid metabolite in human carcinoma. Second, the in vitro validation of the chemodesensitising effect of PE O– demonstrated a possible cause for the results of the database analyses. Third, the cellular production of PE O– is more likely to facilitate EOC progression than PE, indicating a dependence on ether lipid metabolism.

### 4.1 Interdependence Between Lipid Resources and Cancer Development

Although there have been successes in demonstrating lipid biosignatures in cancer chemoresistance, an unbiased systemic evaluation of lipid metabolites is still lacking. The difficulties may be due to the absence of advanced lipid profiling technology in omics and at a high throughput scale [40]. Since lipidomic technology has progressed rapidly in the past two decades [41], this issue has become testable. In addition, endogenous production or exogenous resources may contribute to the disturbed lipid homeostasis in cancer. Cancers could be bypassed if either one of the lipid resources is blocked. For example, in steroid hormone homeostasis, cholesterolgenesis and steroidogenesis genes have varied impacts on gastric cancer. Cholesterolgenesis gene expression does not have an effect on the prognosis of gastric cancer patients [42]. However, steroidogenesis gene expression is a promoter of cancer progression [27]. While cholesterol is the substrate of steroidogenesis, its importation via lipoprotein receptors has been shown to promote cancer progression [27]. Another well‐known discrepancy is related to statin (HMG‐CoA reductase inhibitor) use in retrospective observations compared to prospective clinical trials. Statin users are associated with a significantly lower risk of liver cancer in post hoc data analysis [43], but statin use failed to prolong survival in a prospective trial [44].

### 4.2 PE O– Resources in EOC

The sources of glycerol‐backboned phospholipids may be either exogenous or endogenous. Although the glycerol‐backboned phospholipid classes are varied, the glycerol resources are mainly derived from glycerol‐3‐phosphate (G3P) during glycolysis [45]. By the addition of an acyl‐carbon chain or functional group, either neutral lipids, for example, DAG or TAG, or phospholipids (also known as Kennedy's pathway) [46], for example, PI, PC and PE, can be formed. However, neutral lipids and phospholipids can also be imported via lipoproteins and their corresponding receptors. For example, Lee et al. found that atrial remodelling is related to VLDL‐mediated unsaturated fatty acid import, for example, TAG and lysophosphatidylcholine (LPC) or LPE [47].

In the case of the cancer lipidome, our previous studies suggested that the lipid importation route may modulate chemosensitivity [30]. In a comparison of lipidomic profiles, LPC (negatively correlated) and PE O– (positively correlated) were found to be highly related to chemosensitivity in EOC cells [31]. At that time, the role of PE O– had not yet been studied. The speculation that PE O– related to chemoinsensitivity was unexplored. Ethanol fatty acid imported from the environment could be conjugated with the glycerol backbone and then converted by AGPS to form ether lipids [48]. Therefore, the source of abundant PE O– could be both endogenous and exogenous.

### 4.3 The Novel Chemo‐Desensitiser Role of PE O– In EOC Cells

Previous studies have reported significantly higher levels of ether lipids in human tumours compared to normal tissues, and subsequent research has confirmed elevated ether lipid content in various cancer cells and tumours, correlating with their proliferative and tumorigenic potential [38]. In another study examining serum lipid markers in an EOC cohort, Salminen et al. found that PC O– was an indicator of EOC, but its source was not identified [49]. To date, no study has reported chemosensitivity or chemoresistance mechanisms involving ether lipids. A previous report of a synthetic ether PC analogue indicated chemosensitivity through AKT activation [50], which is a finding opposite to that of this study. Therefore, this study's finding is novel, and the relevant molecular mechanism deserves in‐depth study in the future.

## 5 Conclusion

As a lipid marker of chemoinsensitivity in human carcinoma cell lines and in EOC subtypes, the performance of PE O– indicates an innate mechanism against chemoagent challenge in cancer cells. The ether lipid‐related chemosensitivity mechanism indicates a dominant biological event in carcinoma.

## Author Contributions

**Yu‐Ting Su:** conceptualization (equal), formal analysis (equal), writing – original draft (equal). **Wei‐Chun Chang:** data curation (equal), formal analysis (equal), funding acquisition (equal), investigation (equal), methodology (equal), resources (equal), validation (equal), writing – original draft (equal). **Lumin Chen:** data curation (equal), investigation (equal), methodology (equal), validation (equal), writing – original draft (equal). **Ying‐Chun Yu:** investigation (equal), methodology (equal). **Wen‐Jen Lin:** methodology (equal). **Jheng‐You Lin:** formal analysis (equal). **Wei‐Chung Cheng:** funding acquisition (equal), investigation (equal), resources (equal). **Juan‐Cheng Yang:** data curation (equal), funding acquisition (equal), resources (equal). **Yao‐Ching Hung:** data curation (equal), funding acquisition (equal), investigation (equal), resources (equal), writing – review and editing (equal). **Wen‐Lung Ma:** conceptualization (equal), funding acquisition (equal), project administration (equal), resources (equal), writing – review and editing (equal).

## Conflicts of Interest

The authors declare no conflicts of interest.

## Supporting information


Table S1.

Table S2.

Table S3.

## Data Availability

All data generated or analysed during this study are included in this published article and its Tables [Link], [Link].
