# Supplementary material for: Ether‐Linked Glycerophospholipids Are Potential Chemo‐Desensitisers and Are Associated With Overall Survival in Carcinoma Patients
Source: J Cell Mol Med. 2024 Dec 19;28(24):e70277. doi: 10.1111/jcmm.70277 (PMC11657596; doi:10.1111/jcmm.70277)
Supplement: Supplementary file 1 — Table S1. [file JCMM-28-e70277-s002.pdf]

Supplementary Table 1. Cell culture media.

Carcinoma type	Cell line	medium
EOC	OVCAR3	Dulbecco's modified Eagle's medium
	SKOV3ip1	Dulbecco's modified Eagle's medium
	MDAH-2774	Dulbecco's modified Eagle's medium
	TOV-112D	105:199 medium
	TOV-21G	105:199 medium
	ES-2	McCoy's 5a Medium
	HeyA8	RPMI-1640 Medium
	OVCAR429	Dulbecco's modified Eagle's medium
	SKOV3	Dulbecco's modified Eagle's medium
HCC	Tong	Dulbecco's modified Eagle's medium
	Huh7	Dulbecco's modified Eagle's medium
	HCC36	Dulbecco's modified Eagle's medium
	SK-HEP-1	Eagle's Minimum Essential Medium
	HepG2	Dulbecco's modified Eagle's medium
	Mahlavu	Dulbecco's modified Eagle's medium
	HEP3b	Dulbecco's modified Eagle's medium
RCC	RCC42	RPMI-1640 Medium
	786-O	RPMI-1640 Medium
	769-P	RPMI-1640 Medium
	OS-RC-2	RPMI-1640 Medium
	Caki-1	RPMI-1640 Medium
	A-498	Eagle's Minimum Essential Medium
GCa	AGS	RPMI-1640 Medium
	SC-M1	RPMI-1640 Medium
	MKN45	RPMI-1640 Medium
CC	SSP25	RPMI-1640 Medium
	BDE	Dulbecco's modified Eagle's medium
	HuCCT1	RPMI-1640 Medium
	RBE	RPMI-1640 Medium
	H1	RPMI-1640 Medium
PDAC	PANC-1	Dulbecco's Modified Eagle's Medium
	MIA PaCa-2	Dulbecco's Modified Eagle's Medium
	AsPC1	RPMI-1640 Medium
	BxPC3	RPMI-1640 Medium
HNPC	SAS	RPMI-1640 Medium
	FaDu	Eagle's Minimum Essential Medium
	SCC-4	DMEM/F12 Medium
	SCC-25	DMEM/F12 Medium
	Detroit 562	Eagle's Minimum Essential Medium

Carcinoma type	Cell line	medium
CCa	HCT 116	McCoy's 5a medium
	LS 180	Eagle's Minimum Essential Medium
	HT-29	McCoy's 5a medium
	DLD-1	RPMI-1640 Medium
	HCT-8	RPMI-1640 Medium
	COLO 320DM	RPMI-1640 Medium
ADLC	A549	RPMI-1640 Medium
	H1299	RPMI-1640 Medium
	H441	RPMI-1640 Medium
BLCA	NTUB1	RPMI-1640 Medium
	T24	McCoy's 5a Medium
	J82	Eagle's Minimum Essential Medium
	TCCSUP	Eagle's Minimum Essential Medium
	BFTC905	RPMI-1640 Medium
	5637	RPMI-1640 Medium
	SV-HUC-1	F-12K Medium
	TSGH-8301	RPMI-1640 Medium
	HT-1197	Eagle's Minimum Essential Medium
	HT-1376	Eagle's Minimum Essential Medium
RCC	BFTC909	Dulbecco's modified Eagle's medium
	HK-2	Keratinocyte Serum Free Medium
	MC-SV-HUC T-2	Ham's F12 medium
