# Supplementary material for: Ether‐Linked Glycerophospholipids Are Potential Chemo‐Desensitisers and Are Associated With Overall Survival in Carcinoma Patients
Source: J Cell Mol Med. 2024 Dec 19;28(24):e70277. doi: 10.1111/jcmm.70277 (PMC11657596; doi:10.1111/jcmm.70277)
Supplement: Supplementary file 2 — Table S2. [file JCMM-28-e70277-s001.pdf]

Supplementary Table 2. List of genes associated with glycerophospholipid metabolism

lipid-related pathways	Lipid-related genes
hsa00564_Glycerophospholipid_metabolism	GPD1L, GPD1, GPD2, GPAM, GPAT2, GPAT4, GPAT3, AGPAT1, AGPAT2, AGPAT5, LCLAT1, MBOAT1, MBOAT2, AGPAT3, AGPAT4, GNPAT, ADPRM, PLPP1, PLPP3, PLPP2, LPIN1, LPIN3, LPIN2, PLPP5, PLPP4, DGKZ, DGKD, DGKI, DGKA, DGKE, DGKB, DGKH, DGKG, DGKQ, DGKK, CHPT1, CEPT1, PLD1, PLD2, PLD3, PLD4, LCAT, PLA2G10, PLA2G2D, PLA2G2E, PLA2G3, PLA2G2F, PLA2G12A, PLA2G12B, PLA2G1B, PLA2G5, PLA2G2A, PLA2G2C, PLA2G4E, PLA2G4A, JMJD7-PLA2G4B, PLA2G4B, PLA2G4C, PLA2G4D, PLA2G4F, PLA2G6, PLB1, PLA2G16, LPCAT2, LPCAT1, LPCAT4, LPCAT3, LYPLA1, PLA2G15, LYPLA2, PNPLA6, PNPLA7, GPCPD1, CHAT, ACHE, CHKA, CHKB, PHOSPHO1, PCYT1B, PCYT1A, SELENOI, ETNK1, ETNK2, PCYT2, ETNPPL, PEMT, CDS1, CDS2, PTDSS1, PTDSS2, PISD, PGS1, CRLS1, TAZ, LPGAT1, CDIPT, MBOAT7
