# Supplementary material for: Ether‐Linked Glycerophospholipids Are Potential Chemo‐Desensitisers and Are Associated With Overall Survival in Carcinoma Patients
Source: J Cell Mol Med. 2024 Dec 19;28(24):e70277. doi: 10.1111/jcmm.70277 (PMC11657596; doi:10.1111/jcmm.70277)
Supplement: Supplementary file 3 — Table S3. [file JCMM-28-e70277-s003.pdf]

Supplementary Table 3. List of cisplatin- and LPC-cisplatin-sensitive and resistant cell lines.

	cisplatin	LPC+cisplatin
sensitive	OVCAR3	MDAH-2774
	TOV-21G	769-P
	SKOV3	AGS
	RCC42	BxPC-3
	786-O	AsPC-1
	OSRC-2	CFPAC-1
	Caki-1	HepG2
	A498	
	SCM1	
	MKU45	
	BDE	
	HuCCT1	
	H1	
	PanC1	
	MIACapa-2	
resistance	PLC5	TONG
	SKhep1	SKhep1
	SSP25	SSP25
	RBE	RBE
	BxPC-3	
